# Human Neural Progenitor Cell Engraftment Increases Neurogenesis and Microglial Recruitment in the Brain of Rats with Stroke

**DOI:** 10.1371/journal.pone.0050444

**Published:** 2012-11-21

**Authors:** Zahra Hassani, Joanna O'Reilly, Yewande Pearse, Paul Stroemer, Ellen Tang, John Sinden, Jack Price, Sandrine Thuret

**Affiliations:** 1 Centre for the Cellular Basis of Behaviour, The James Black Centre, King's College London, Institute of Psychiatry, London, United Kingdom; 2 ReNeuron Group plc, Guildford, Surrey, United Kingdom; University of South Florida, United States of America

## Abstract

**Main Objectives:**

Stem cell transplantation is to date one of the most promising therapies for chronic ischemic stroke. The human conditionally immortalised neural stem cell line, CTX0E03, has demonstrable efficacy in a rodent model of stroke and is currently in clinical trials. Nonetheless, the mechanisms by which it promotes brain repair are not fully characterised. This study investigated the cellular events occurring after CTX0E03 transplantation in the brains of rats that underwent ischemic stroke.

**Methods:**

We focused on the endogenous proliferative activity of the host brain in response to cell transplantation and determined the identity of the proliferating cells using markers for young neurons (doublecortin, Dcx) and microglia (CD11b). So as to determine the chronology of events occurring post-transplantation, we analysed the engrafted brains one week and four weeks post-transplantation.

**Results:**

We observed a significantly greater endogenous proliferation in the striatum of ischemic brains receiving a CTX0E03 graft compared to vehicle-treated ischemic brains. A significant proportion of these proliferative cells were found to be Dcx+ striatal neuroblasts. Further, we describe an enhanced immune response after CTX0E03 engraftment, as shown by a significant increase of proliferating CD11b+ microglial cells.

**Conclusions:**

Our study demonstrates that few Dcx+ neuroblasts are proliferative in normal conditions, and that this population of proliferative neuroblasts is increased in response to stroke. We further show that CTX0E03 transplantation after stroke leads to the maintenance of this proliferative activity. Interestingly, the preservation of neuronal proliferative activity upon CTX0E03 transplantation is preceded and accompanied by a high rate of proliferating microglia. Our study suggests that microglia might mediate in part the effect of CTX0E03 transplantation on neuronal proliferation in ischemic stroke conditions.

## Introduction

Stroke is the third major cause of death and the single major source of disability in developed countries. Ischemic stroke represents 87% of the total [1], and has debilitating consequences ranging from motor function impairments to complete paralysis. The only treatment for ischemic stroke so far is the administration of tissue plasminogen activator (TPA) within three hours after the onset of the stroke [2]. However, only 8% of the patients diagnosed with an ischemic stroke are eligible for TPA treatment [1], leaving a substantial need for the development of alternative therapies. Stem cell transplantation may have the potential to address this unmet medical need.

A promising therapeutic approach involves the transplantation of human immortalised neural stem cell lines, such as the CTX0E03 cell line. CTX0E03 were derived from human first trimester fetal cortical cells. These clinical grade cells have been genetically modified with the c-mycER(TAM) technology to achieve conditional growth control with a fusion protein comprising a growth promoting gene, c-myc, and a hormone receptor regulated by the synthetic drug, 4-hydroxy-tamoxifen (4-OHT) [3]. The CTX0E03 cells promote robust recovery of motor function after transplantation into the brain of ischemic rats (ischemia induced by middle cerebral artery occlusion, MCAO), in a dose-dependent manner [4]. However, the mechanism of action appears not to be cell replacement, since very few cells survive after transplantation [4] and few graft-derived cells differentiate as neurons or glia [5]. Rather, engrafted cells of this type seem to improve cerebral blood flow after stroke [6] and appear to modulate the host response to injury, thereby producing presumably a neurotrophic or disease-modifying effect. The CTX0E03 cell line recently entered a non-randomised, single-dose phase I clinical trial (PISCES), aiming at assessing their safety after transplantation in the brain of male patients who remained disabled after 6 months to 5 years following an ischemic stroke and aged over 60 years old. Details on this trial (NCT01151124), which is currently recruiting patients, can be found on the clinical trials database (clinicaltrial.gov). Given the clinical prospects carried by CTX0E03, the need to understand better this mode of action is thus critical.

A previous longitudinal study examined the effect of engrafting a similar mouse cell line into rat MCAO [7]. It demonstrated a correlated impact of engraftment on both sensorimotor behaviour and striatal brain structure that was detectable after four weeks. The CTX0E03 cells appear to work over a similar time course [3]. Many of the cellular or molecular effects of stem cell engraftment following stroke have been observed at relatively short time points. In this study, we aimed to discover cellular therapeutic correlates over a longer four-week time course after transplantation of CTX0E03 cells into the striatum of MCAO rats. We present here evidence for a pool of endogenous proliferating cells generated in response to stroke and engraftment. We show that this pool is composed of neuroblasts and of microglial cells, and suggest that the microglial effect might precede the impact on neurogenesis.

Interestingly, our study highlights a very small population of cells, the Ki67/Dcx double-positive cell population, extremely rare in the striatum of sham animals [8]. We found that stroke strongly activates this population of proliferating neuroblasts, and that the engraftment of CTX0E03 cells maintains this proliferative rate for a longer time period than in the vehicle-transplanted control animals. This protective effect of CTX0E03 over the reactive proliferative neuroblast population could contribute to the therapeutic effect of CTX0E03.

## Materials and Methods

### Animals

All animal procedures were performed under the Project Licence number 70/14472 and complied with the UK Animals (Scientific) Procedures Act (1986) and the Ethical Review Process of King's College London. All surgeries were performed under isoflurane anesthesia, and all efforts were made to minimize suffering. Animals (320/360 g male CD rats, purchased from Charles River) were maintained under standard housing conditions.

### MCAO surgery and cell transplantation

Groups of six to eight rats were used for the study. In the MCAO groups, as described previously [4], a coated filament (Doccol) was introduced into the middle cerebral artery of anaesthetised (Isoflurane 3%, Merial, UK) animals via the common carotid artery and allowed to recover from anaesthesia. Occlusion was confirmed by evaluation of dysfunction in the whiskers test and circling behaviour [4] at approximately 30 minutes after the onset of occlusion. Rats were re-anaesthetized at approximately 55 minutes after the onset of occlusion and the filament retracted at 60 minutes, restoring the blood flow. All animals were given rehydration therapy of 2 mL Duphalyte (Fort Dodge, Southhampton, UK) in 3 mL glucose saline (IVEX) on the first 2 days of recovery from occlusion surgery. The efficiency of the surgery was assessed the following morning and the following week of recovery by whiskers test and the use of a neurological scoring battery [7]. Only animals showing a sustained dysfunction were included in the study (e.g. loss of reflexive paw placement in the whiskers test). The sham groups received the same surgery but the filament was pushed into the artery for only 5 mm in depth. The filament was then cut and left inside the artery, and the animals were sutured. This short length of filament does not reach the middle cerebral artery, so no occlusion occurred in this group of animals.

The rats were returned to their housing for recovery during 4 weeks. By this time, most of the rats had recovered their pre-operation weight. Four weeks after MCAo, engraftment of CTX0E03 cells was performed. The myc-ER^TAM^ CTX0E03 human neural stem cell line has been described previously [3]. The cells were resuspended into N-acetylcysteine (NAC) at a concentration of 50,000 cells/µl. Two separate injections (4.5 µl of cell suspension per injection) were performed per animal, into the two following sites from Bregma: Anterior −1.3 mm, Lateral right 3.5 mm, Ventral −6.5 mm for site 1 and Anterior −1.8 mm, Lateral right 4.0 mm, Ventral −6.0 mm for site 2. Thus, a total of 450,000 cells were injected per animal. The volumes were administered slowly (4.5 µl/5 min) and the needle was left for a further 4 minutes before withdrawal. In the control groups, NAC without cells was injected, following the same protocol. All animals were given rehydration therapy of 2 mL Duphalyte in 3 mL glucose saline (IVEX) on the first 2 days of recovery. All animals received 20 mg/kg methylprednisolone (Solu- Medrone; Pharmacia, Milton Keynes, UK) subcutaneously, with 1 dose 24 hours prior to either cell or vehicle implantation, 1 dose at implantation and daily for 2 weeks, and 10 mg/kg cyclosporine-A (CsA; Sandoz Pharmaceuticals, East Hanover, NJ) in Cremophor EL (Sigma) carrier subcutaneously with 1 dose 24 hours prior to cell implantation, 1 dose at implantation, and then 6 doses over 2 weeks. Depending on the time point, the animals were sacrificed 1 or 4 weeks post-transplantation by sodium pentobarbital. Each rat was perfused with a heparinized saline solution (NaCl 0.9%) followed by 4% cold paraformaldehyde fixative (Pioneer Chemicals,UK) (4°C). The brains were dissected, washed three times in PBS, then kept at 4°C in 30% sucrose.

### Immunostainings

Free-floating 50 µm-coronal-sections were conserved at −20°C in 12 wells plates, in a cryoprotective solution.

Ki67 DAB staining was performed using the ABC/Elite kit solution from Vector labs, according to the manufacturer's instructions. The primary antibody (Ki67 SP6 NEOMARKERS) was used overnight at 1/200 dilution. After dehydration, the sections were mounted in DPX (Sigma).

For fluorescent immunostainings, the following primary antibodies were used: Mouse anti-CD11B (Chemicon, 1/400); Goat anti-DCX (Abcam, 1/500) and Rabbit anti-KI67 (Fremont, 1/250) in TBST overnight at 4°C. Secondary detection was performed with: Donkey anti–Goat Alexa 594 nm, anti-Rabbit Alexa 488 nm and anti-mouse Alexa 647 nm or 680 nm. All secondary antibodies were used at 1/500. After washings, the sections were coverslipped in Prolong Gold (Invitrogen).

### Cell counting and statistical analyses

Cell countings were done in blind conditions. Stereology (Optical Fractionator) was used as described previously [9] to count the number of Ki67 stained cells in all conditions to minimise bias and optimise reliability. Ki67-positive cells were counted in 3 separate areas: the SVZ, the dorsal striatum lining the corpus callosum and the striatal parenchyma. Cells were counted on the ipsilateral side to the lesion of MCAo brains and on one side of the Sham brains in 7 mounted sections starting where the lateral ventricles first become visible by eye. These sections were in intervals of one-in-twelve series of sections therefore 600 µm apart, starting with the most anterior and progressing posteriorly, throughout the rostrocaudal extent of the granule cell layer of the SVZ. Using an Axioskop 2 MOT Zeiss microscope with a x2.5 objective lens (Zeiss, Germany), a semiautomatic stereology system (StereoInvestigator, Microbrightfield) was used to trace the area of interest and a x40 magnification (Leitz) was used to count the number of cells stained. For each section the computer randomly placed a 20 µm by 20 µm counting frame in different areas of the traced SVZ (grid size of X: 30 µm and Y: 190 µm) with a set number of average sampling sites of 30. Cells that were within the counting frame or touching the green line were counted and marked with a cross. The optical fractionator estimated the total number of Ki67 positive cells by relating the number counted in the random counting frames to the sectional volume and then multiplying it by the reference volume. All stereological cell counts had a Gundersen Coefficient of error ≤0.1 [10].

Confocal microscopy was used to assess co-labelling of Ki-67 and DCX or Ki-67 and CD11b. A 1-in-12 series of sections was double-labeled as described earlier and analyzed by confocal microscopy (Leica TCS SP5, Germany). For each animal, one hundred Ki67- positive cells randomly selected in the whole striatum were analyzed for co-expression of Ki-67 and DCX or CD11b to assess the phenotype, and ratios of cells co-expressing Ki-67 and DCX or CD11b were determined.

All data was analysed using a one-factor ANOVA. Post hoc analysis was carried out using Bonferroni-corrected individual comparisons (Microsoft Excel and XLSTAT; Addinsoft, NY). In all analyses, a P-value of less than 0.05 was chosen as significance threshold. All data are presented as the mean±standard error of the mean (SEM).

## Results

Two experimental protocols were designed (Figure S1). In Protocol 1 (Figure S1A), two groups of rats were prepared (n = 6). Both groups received MCAO surgery. Four weeks post-surgery, animals received either CTX0E03 grafts or vehicle (N-acetyl cysteine solution: NAC), and the animals were sacrificed one week post-transplantation. The second protocol (Figure S1B) had four groups of animals (n = 6–8). One group underwent MCAO surgery followed four weeks later by CTX0E03 engraftment. A second group also underwent MCAO surgery, but received only vehicle injection after four weeks. Groups three and four were similar, but received sham surgery followed by either cell or vehicle engraftment. All four groups were sacrificed 4 weeks post-transplantation, and tissue processed for immunostaining analyses. In the MCAO groups of both protocol 1 and 2, the MCAO surgery generated large lesions (cortical and striatal tissue loss) in the left hemisphere (Figure S1C).

### Early effects of the engrafted cells

**Figure 1:**
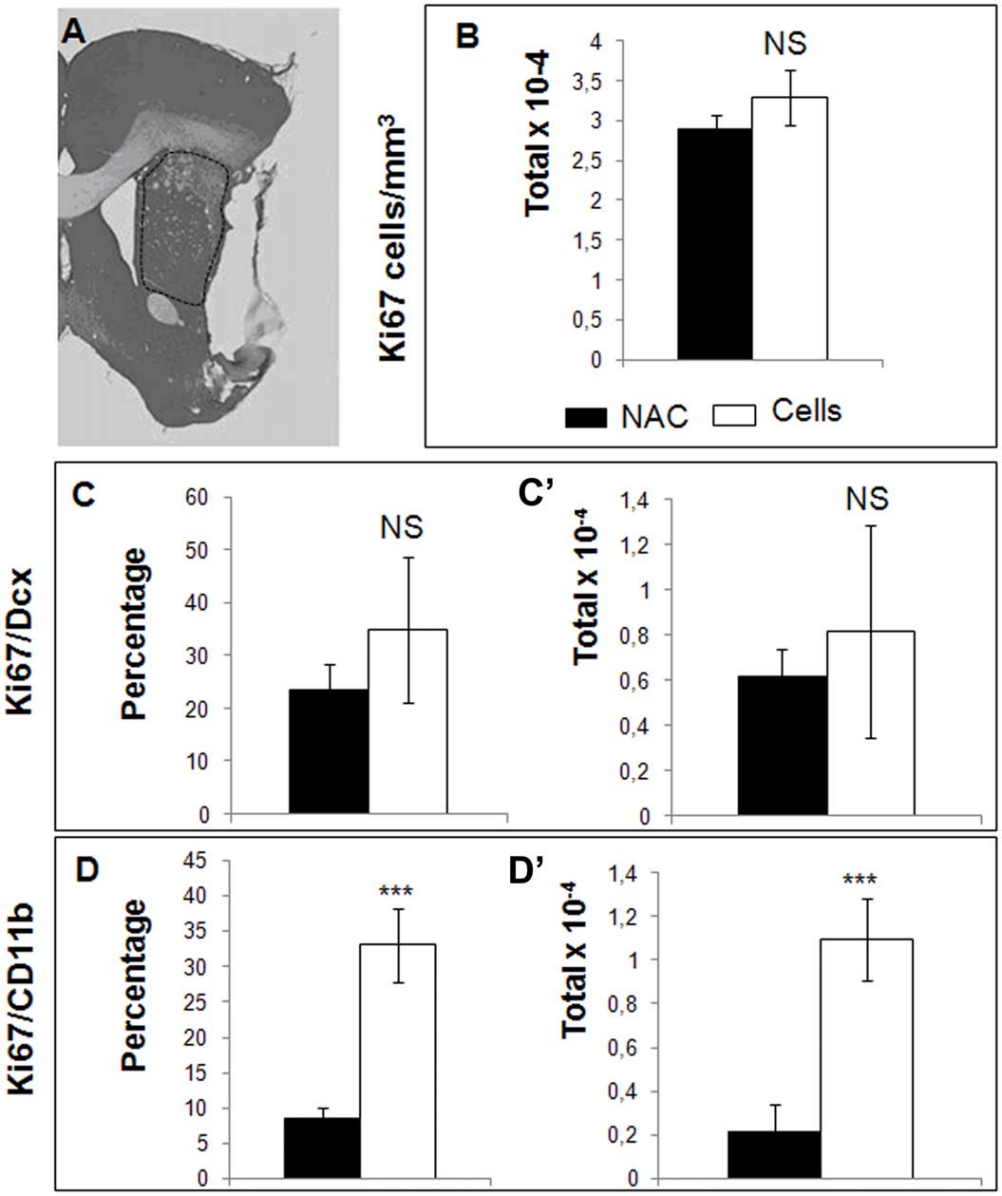
Effect of CTX0E03 one week post-transplantation. A) Ki67+ cells were counted in the ipsilateral striatum (area delineated by a dotted line). Graph B represents the total numbers of proliferative cells per mm^3^ (Ki67-positive) present in the striatum of animals from Protocol 1, calculated with a stereology microscope. C) and D) Left: The percentage of Ki67+/Dcx+ (C) or Ki67+/CD11b+ (D) double-positive cells over the total number of Ki67+ cells is shown; Right: Total numbers of Ki67+/Dcx+ (C) or of Ki67+/CD11b+ (D) cells present in the striatum are shown. NS: not significant; ***: p<0.001.

To investigate the early effects of CTX0E03 on cellular proliferation in MCAO brains, we compared the total numbers of Ki67+ cells in the whole striatum of MCAO/NAC versus MCAO/cells one week post-transplantation. An illustration of the area of interest is shown in Figure? 1A. We found no difference between the two groups (Figure? 1B, 28970 (+/−1747) versus 32958 (+/−3482) proliferative cells per mm^3^). Thus five weeks after the stroke (and one week after engraftment), there are similar numbers of dividing cells in the striatum whether CTXOE03 cells were engrafted or not. To identify the Ki67^+^ cells, we co-labelled with the neuroblast marker, Dcx, and with the microglial marker, CD11b. We found that about 30% of the proliferating cells were Dcx+ (Figure 1C, left), but that cell engraftment had no significant effect on this population at this time point (Figure 1C, right: no statistical significance between MCAO/NAC: 6154 (+/−1226) and MCAO/cells: 8155 (+/−4708) cells per mm^3^). We also found, however, that a proportion of the Ki67^+^ cells were CD11b^+^ microglia, and that CTX0E03 transplantation increased this population four-fold (Figure? 1D, left: 8 (+/−1.4)% versus 33 (+/−5.2)%, respectively). Thus, the most visible effect of CTX0E03 one week post-engraftment is a dramatic increase in proliferating microglia, expressed either as absolute number of cells or as a proportion of the totalKi67^+^ population.

### Later effects of CTX0E03

The same markers (Dcx and CD11b) were used to stain the sections from protocol 2.

#### Increased proliferation in the MCAO/cells group

**Figure 2:**
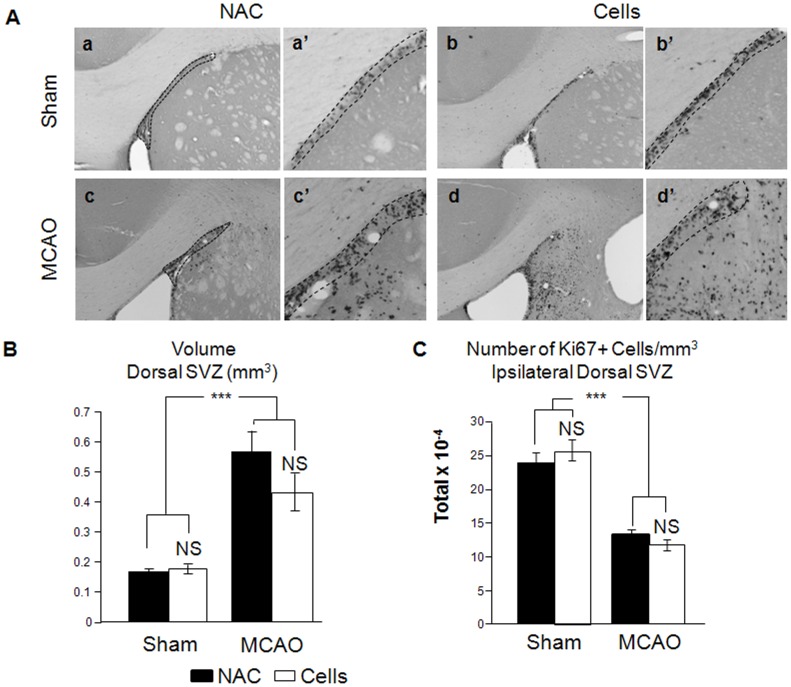
Stroke affects the organisation of the dorsal tail of the SVZ. A) Pictures of the four groups of animals from Protocol 2 are represented (a–d). Pictures in a′, b′, c′ and d′ represent a magnification of the dorsal SVZ from a, b, c and d respectively. B) The volume of the dSVZ was evaluated for each group by stereology; C) The density of Ki67+ cells is shown for each group. NS: Not significant; **: p<0.01; ***: p<0.001.

Activation of proliferation in the subventricular zone (SVZ) in response to stroke has previously been reported [11], [12]. To determine whether CTX0E03 influenced NSPC proliferation in the SVZ, we counted and compared the total number of Ki67+ cells in the SVZ. We counted separately the proliferative cells present in the SVZ lining the lateral ventricles (ventral SVZ (vSVZ), Figure S2B) and those present in the dorsal part of the SVZ, ventro-lateral to the corpus callosum (dSVZ, Figure S2B) in the four conditions described in Figure S1B. We also evaluated by stereology the volume of these two proliferative areas; each area was defined and delineated by a high density of Ki67-positive cells, clearly distinctive from the neighbouring striatum which has a poor density of Ki67+ cells (Figure 2A).

We found no difference in the number of Ki67-positive cells in the vSVZ between the four groups (Figure S3A). The volume of the vSVZ was not affected either by stroke or by the transplanted cells (Figure S3B), and consequently the number of Ki67+ cells per volume in the vSVZ (density of the Ki67+ cells in the vSVZ) was similar in the four groups (Figure S3C).

By comparison, however, the dorsal SVZ was disrupted in response to stroke (Figure 2). There was an increase in the volume of this region in the two MCAO groups (Figure? 2A, c–d′ and Figure? 2B) as compared to the sham groups (Figure? 2A, a–b′ and Figure? 2B) and a decreased density of Ki67+ (Figure? 2C). These data suggest a dispersion of the proliferative pool in the dSVZ of the MCAO groups, with no further effect of the CTX0E03 cells engraftment (Figure? 2B and 2C, no statistical difference between MCAO/NAC and MCAO/cells). We conclude here that the main effects observed on the SVZ (vSVZ and dSVZ) four weeks post-engraftment are due to the stroke event rather than the engraftment of CTX0E03 cells. Indeed, CTX0E03 cells seem to play a minimal role on progenitor proliferation in the ventral and dorsal SVZ, whereas stroke leads to increased proliferation and to the disorganisation of the dorsal SVZ.

We then counted the Ki67^+^ cells in the striatal parenchyma, and observed an increase in the density of Ki67^+^ cells in MCAO/cells group compared to the three other groups (Figure? 3). MCAO causes a doubling in the density of KI67^+^ cells in the striatum, five weeks following the lesion (Figure? 3B, 4801 (+/−684) versus 2435 (+/−873) cells/mm^3^). Engrafting CTX0E03 cells into sham rats also gives a similarly modest non-significant increase (5223 (+/−1346) versus 2435 (+/−873) cells/mm^3^). Engrafting cells into animals that had been subjected to MCAO, however, gives a greater than eight-fold increase in the density of dividing cells (21456 (+/−4364) versus 2435 (+/−873) cells/mm^3^, p<0.001). Thus, in the context of a stroke, engrafting causes a dramatic increase in the number of dividing cells in the striatum at this time point.

**Figure 3:**
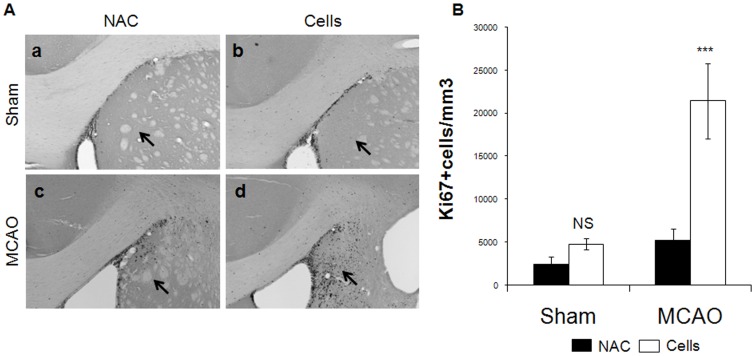
CTX0E03 increase the number of proliferative cells in the striatum four weeks post-transplantation. A) Representative pictures from the four groups of Protocol 2 are shown. A strong increase of Ki67 immunostaining was observed in the striatum of animals from the MCAO/cells group (arrows). B) The total numbers of Ki67+ cells per mm^3^ in the striatum are presented. A statistically significant increase of Ki67 immunostaining was observed in the MCAO/cells group. ***: p<0.001.

#### MCAO favours neurogenic proliferation

We again used Dcx as a marker for neuroblasts, and CD11b for microglial cells to identify Ki67^+^ dividing cells (Figure? 4). Just as for the Ki67^+^ population as a whole, we observed a dramatic increase in the number of Dcx^+^/Ki67^+^ cells (Figure? 4B, top graph). Smaller non-significant increases were seen as a result of MCAO, and as a result of grafting into sham animals, but the corpus striata that received strokes and cell grafts had a more than thirty-fold increase in the density of dividing neuroblasts within the Ki67+ population. The percentage of Dcx+ cells within the proliferative (Ki67+) population (Figure? 4B, bottom graph) was doubled in the two MCAO groups (30.2 (+/−7.7)% and 29.1(+/−7.7)%) compared to the Sham controls groups (12.7 (+/−5.4)% and 11.1(+/−7.5)%, showing that stroke leads to an activation of neuroblast proliferation. This proportion of proliferating Dcx+ cells was not affected by CTX0E03 engraftment (no significant difference between NAC and Cells groups in both Sham and MCAO situations). We then related these ratios to the total number of Ki67 cells (shown in Figure? 3B), and found that the total number of proliferating neuroblasts in the MCAO/cells group (Figure? 4B, top graph) was greatly enhanced (4071 (+/−1367) cells/mm^3^) compared to the MCAO/NAC, Sham/NAC and Sham/cells groups (599 (+/−272) cells/mm^3^, 129 (+/−62) cells/mm^3^ and 736(+/−315) cells/mm^3^, respectively).

**Figure 4:**
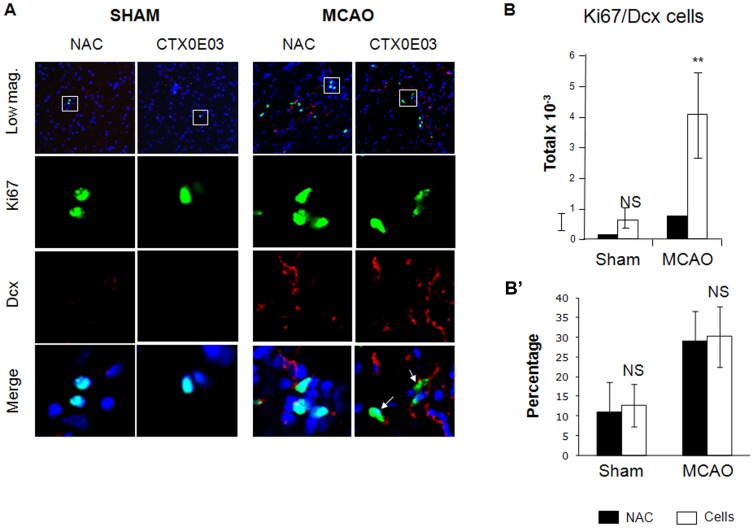
CTX0E03 engraftment increases the number of proliferating neuroblasts in the striatum of stroke animals. A) Immunohistochemistry for Ki67 (green) and Dcx (red) was performed on brain sections from each group (Protocol 2). Some Dcx+ neuroblasts were found to be proliferating, in particular in the MCAO/cells group (arrows). B) Top: The total numbers of Ki67+/Dcx+ cells per mm^3^ are represented. A strong increase in the number of proliferative neuroblasts in the MCAO/cells group is observed; Bottom: The percentage of Dcx+ cells within the proliferative (Ki67+) population is represented for each group. The MCAO condition leads to an increased proportion of neuroblasts within the proliferative pool of cells present in the striatum. NS: not significant; **: p<0.01.

#### Effects of CTX0E03 on microglia

In order to determine the effects of CTX0E03 on microglial proliferation after 4 weeks (protocol 2; Figure S1B), we compared the number of dividing CD11b-positive microglia/macrophages cells in the four animal groups (Figure? 5A). CD11b-positive microglia present in the striatum (illustrated in Figure S2A) were counted by stereology. Just as the MCAO/cells group had a synergistic increase in the number of Dcx+ neuroblasts, so there was a dramatic synergistic increase in the numbers of CD11b+ microglia (Figure? 5B, upper graph). There were more than 6 times the number of CD11b+/Ki67+ cells in the MCAO/cells group (4283 (+/−2158) cells/mm^3^) compared to the SHAM/cells group (682 (+/−440) cells/mm^3^), and more than 14 times compared to the MCAO/NAC group (288 (+/−91) cells/mm^3^). We observed a larger proportion of Ki67 cells expressing the microglial marker CD11b (Figure? 5B, lower graph) in the presence of CTX0E03 cells both in the sham and in the MCAO situation. But this increase was statistically significant (p<0.05) only in the MCAO situation and not in the Sham groups (sham/NAC and sham/cells groups not statistically different).

**Figure 5:**
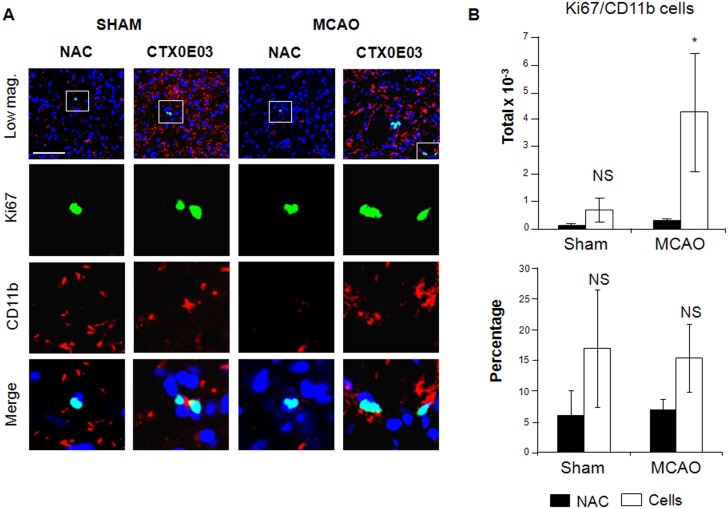
CTX0E03 lead to microglial proliferation, regardless of stroke. A) Immunohistochemistry for Ki67 (green) and CD11b (red) were performed. A visible increase of microglial staining (CD11b) can be observed in the Sham/cells and MCAO/cells, as compared to the Sham/NAC and MCAO/NAC groups (low magnification pictures). B) Quantification of the total number (top graph) and relative proportion (bottom graph) of proliferative microglia (Ki67/CD11b double-positive cells) are represented. More proliferating microglial cells were found in the MCAO/cells groups as compared to the other conditions. NS: not significant; *: p<0.05.

These data show that CTX0E03 engraftment leads to an increased proliferation of microglial cells into the host parenchyma, and that the rate of microglial cell division is enhanced in the ischemic context.

### CTX0E03 cells maintain stroke-induced neurogenesis

In order to visualise the effect of CTX0E03 post-engraftment over time, we plotted the numbers of dividing neuroblasts (Ki67+/Dcx+) and dividing microglia (Ki67+/CD11b+) at one week and four weeks post-engraftment within a single longitudinal graph (Figure? 6). This figure shows that the overall proliferative activity into the striatum (induced by stroke) decreases over time. Engraftment with CTX0E03 cells does not have a significant effect on this proliferation at one week, but engraftment does slow this reduction observed over the four weeks (Figure? 6A). Proliferative Dcx+ neuroblasts reflect this overall pattern: the reduction of these cells over time is reduced by CTX0E03 engraftment (Figure? 6B). The Ki67+/CD11b+ microglia show a different pattern, however. These cells are dramatically increased at just 1 week following graft compared to the vehicle-injected group, and the difference is still significant at 4 weeks (Figure? 6C).

**Figure 6:**
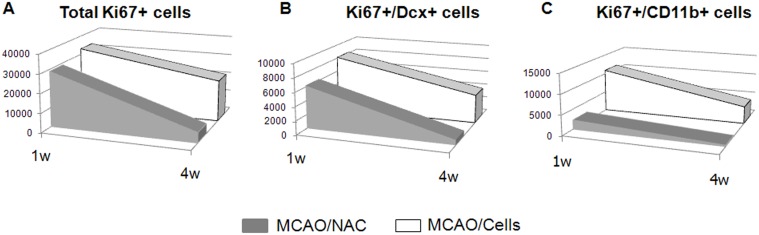
Representation of the total number of proliferative cells at 1 week and 4 weeks post-transplantation. Total numbers of Ki67+ (A), Ki67+/Dcx+ (B) and Ki67+/CD11b+ (C) cells per mm^3^ in the striatum are shown for MCAO/NAC and MCAO/cells groups, one week and four weeks post-transplantation. A) In the absence of cells, the number of proliferative cells in the striatum decreased dramatically between the two time-points (grey volume). In the presence of CTX0E03, the number of Ki67 cells in the striatum decreased more slowly (white volume). B) As for the total number of Ki67+ cells, the numbers of proliferating neuroblasts was maintained by the presence of CTX0E03 (white) as compared to the loss observed in NAC-injected animals (grey). C) Only few microglial cells proliferate at the two time points observed (grey), whereas CTX0E03 cells lead to increased microglial proliferation (white) that decreases with time.

## Discussion

Stem cell therapies were originally conceived for neurodegenerative disorders because of the potential to replace lost brain cells. Increasingly, however, they are observed to have other modes of action. In the short term, these might be more beneficial than true brain reconstruction [13]. CTX0E03 is a neural stem cell line currently in clinical development for stroke [3]. This present study attempts to uncover the mode of action of this cell line. We have examined the impact of CTX0E03 engraftment on dividing (Ki67+) cells in the host striatum. We demonstrate two effects. First, engraftment significantly increases the number of Dcx+ dividing neuroblasts found in the striatum. This effect is not significant one week following the graft, but the number of dividing neuroblasts retained at 4 weeks is significantly raised. Second, CTX0E03 engraftment substantially increases the dividing microglial population. This is particularly dramatic at the one-week time point, but is still significant at four weeks. CTX0E03 engraftment affects both these populations of cells, but the magnitude and kinetics of the effect suggest that the primary effect may be on the microglia. Our data are consistent with (though do not prove) a model in which a primary effect on microglia supports a positive mitogenic and/or survival effect on host neuroblasts.

We are not the first to identify an effect of stroke on neurogenensis. Thored *et al* showed that long-lasting neurogenesis takes place in the ischemic brain, as shown by the production and migration of neuroblasts towards the site of injury several months post-injury [12]. The increased proliferation was transient in the ipsilateral SVZ of ischemic animals, and disappeared six weeks post-injury. This is consistent with our observations, as we found no difference in Ki67 staining in the SVZ 8 weeks post-stroke between Sham and MCAO. More recently, Tajiri et al. [14] demonstrated that amniotic fluid-derived stem cells (AFS cells) delivered intravenously in a rat model of stroke had a therapeutic effect on motor and cognitive functions. Interestingly, the authors describe a population of proliferating young neurons (Ki67+/MAP2+ double positive cells) which increases significantly in the dentate gyrus of animals that received AFS cells. This result corroborates with our present demonstration that upon engraftment, a subpopulation of proliferating committed cells can be recruited and activated.

Indeed, most studies have considered post-mitotic neuroblasts following injury, but only few studies have described dividing neuroblasts [8], [15]–[16]. Walker *et al* showed that under normal conditions, a population of neuroblasts expressing low amounts of Dcx (Dcx^low^) remain multipotent and retain the ability to generate neurospheres *in vitro*, unlike their Dcx^high^ counterparts [8]. The authors demonstrate that in the fetal mouse brain (E14.5), as well as in the P2 mouse brain, about 1% of Dcx+ neuroblasts express the proliferation marker Ki67. This work demonstrates that a sub-population of neuroblasts remains plastic.

Another relevant study from Zhang *et al* [17]focused on the proliferating neuroblasts population after stroke. In this work, the authors demonstrate that in response to an ischemic stroke, proliferative Dcx+ neuroblasts from the SVZ migrate laterally towards the lesion, and can be found in the ipsilateral ischemic striatum. Our study indicates that these Dcx+/Ki67+ proliferating neuroblasts present in the ischemic striatum are preferentially recruited by stem cell engraftment following an ischemic event, or that post-mitotic neuroblasts revert to a proliferative profile. This reactivity of the neuroblast population might be worth further investigation, as it could lead to beneficial advances in the context of brain repair therapies.

The impact of microglia on stroke damage is multifaceted. Damage to the brain incites a cascade of events culminating in the activation of microglia, which release a repertoire of pro- and anti- inflammatory molecules to induce a neuroinflammatory response[18]–[22].This attempt to deal with injury removes dead and damaged neurons and re-establishes normal function [23]. But these molecules can also have detrimental effects on the brain, by attenuating the increase in neurogenesis that follows injury [22]–[23]. Microglial inflammation is an acknowledged consequence of ischemic stroke in patients, and several studies describe microglia-released cytokines as detrimental for the post-ischemic brain [24] [11]. Conversely, inflammation can promote progenitor proliferation, survival, migration and differentiation in the brain [25]. It appears that the negative effects of microglial occur during the acute phase of injury, while the long-term accumulation of microglia might increase neurogenesis [25], [26]. After the acute phase of injury, some microglia change into another neuroprotective state marked by a change in the cytokine production [25]. The production of harmful molecules (like TNF-α) is downregulated whereas the effective suppressors of the pro-inflammatory molecules such as IL-10 and Prostagladin E2 (PGE2) are upregulated [27]. Thored and colleagues (2007) showed that at sixteen weeks, 5% of microglia expressed IGF- 1 protein, which can moderate apoptosis and enhance proliferation and differentiation of NSCs in the SVZ [26]. A pivotal paper by Lalancette-Hebert *et al* demonstrated that proliferative microglia is neuroprotective to the ischemic brain, and that selective ablation of proliferative CD11b-positive cells after stroke leads to an increased infart volume [27]. This is consistent with our hypothesis that the positive effect of CTX0E03 cells on motor function recovery might be explained at least partly by the increased levels of proliferative microglia observed after CTX0E03 engraftment. Two papers related to our study are noteworthy here: Capone and colleagues [28] demonstrated that neural stem cells engraftment after stroke has a neuroprotective effect on the brain release of cytokines and trophic factors. Indeed, they show that neurosphere-derived cells engrafted to the brain of rats 4 h after focal ischemia lead to an activation of microglia and macrophages recruitment and release SDF-1a, TGF-b1, VEGF-A, IGF1 and BDNF, and that this activation of microglia is associated with recovery. The other study is the one by Daadi et al (2010), where the investigators engrafted human neural stem cells into the brain of a mouse model of neonatal hypoxic ischemic brain injury [29]. They observed that cell engraftment led to an increased microglial response, associated with an enhancement of axonal sprouting in the host brain. Note worthily, in this last paper, the authors observe as well a strong increase of Dcx 4 weeks post-engraftment of neural stem cells, consistent with our own observations. None of these studies or the data presented here tackled the question of identifying which type of microglia was enhanced in response to stroke or to cell engraftment. Indeed, we studied the effects of CTX0E03 cells engraftment on the proliferation of microglia regardless of their level of activation. An important future direction will be to label proliferative cells for specific molecular/phenotypical markers differentiating these different microglial populations, since they are possibly playing different roles in the progression of brain injury.

CTX0E03 cells have a therapeutic effect in rats following MCAO that can be observed as an improvement in sensori-motor function [3]–[5] [7]. Altogether, these previous studies [27]–[29] support our hypothesis that CTX0E03-mediated recovery in ischemic stroke conditions might involve the neuroprotective action of microglial populations, and involves the recruitment of the proliferative neuroblasts population.

## Supporting Information

Figure S1 **Experimental protocol and illustrative pictures of brain sections.** The two experimental protocols followed in this study are illustrated (Protocol 1 and Protocol 2). A) In the Protocol 1, two groups of animals were generated. All animals received MCAO surgery, and were left for recovery for 4 weeks. After recovery, half of the animals received a NAC stereotaxic injection (control group) and the other half received CTX0E03 cells transplantation. Animals from both groups were sacrificed 1 week post-transplantation ( = 5 weeks post-MCAO surgery). B) In the second protocol, four groups of animals were generated. Half of the animals had a MCAO surgery while the other half were Sham controls. In MCAO and Sham groups, half of the rats received a NAC stereotaxic injection whereas the other half received a cell transplantation. All the animals were sacrificed 4 weeks post-transplantation ( = 8 weeks post-MCAO). C) Representative pictures from each group of Protocol 2 are shown.
(TIF)

Figure S2 **Definition of the areas of countings.** A) All cells counted in the “striatal” area were contained in the dotted area. B) The dorsal SVZ (dSVZ) and ventral SVZ (vSVZ) are shown (black and white dotted lines, respectively).
(TIF)

Figure S3 **Effects of CTX0E03 on SVZ volume and proliferative activity four weeks post-transplantation.** The numbers of Ki67 cells (A), the volume (B) and the density of Ki67 cells (C) in the SVZ of animals from the four groups of Protocol 2 were calculated by stereology. No difference was found in the volume (B) or proliferative activity (A and C) of the SVZ between the four groups. NS: not significant.
(TIF)
