# Supplementary figures and images for: Human Neural Progenitor Cell Engraftment Increases Neurogenesis and Microglial Recruitment in the Brain of Rats with Stroke
Source: PLoS One. 2012 Nov 21;7(11):e50444. doi: 10.1371/journal.pone.0050444 (PMC3503964; doi:10.1371/journal.pone.0050444)

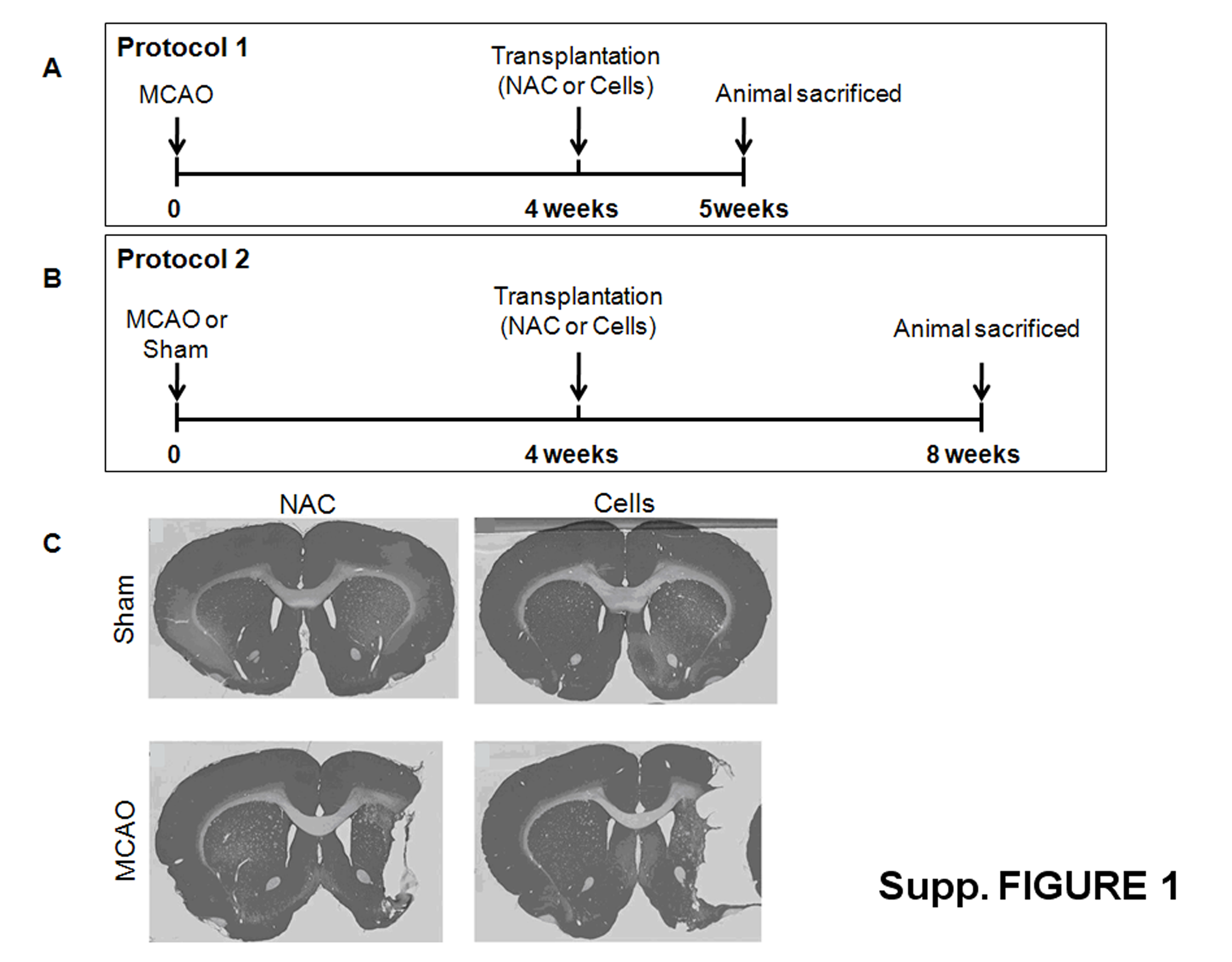

Supplement: Figure S1 — Experimental protocol and illustrative pictures of brain sections. The two experimental protocols followed in this study are illustrated (Protocol 1 and Protocol 2). A) In the Protocol 1, two groups of animals were generated. All animals received MCAO surgery, and were left for recovery for 4 weeks. After recovery, half of the animals received a NAC stereotaxic injection (control group) and the other half received CTX0E03 cells transplantation. Animals from both groups were sacrificed 1 week post-transplantation ( = 5 weeks post-MCAO surgery). B) In the second protocol, four groups of animals were generated. Half of the animals had a MCAO surgery while the other half were Sham controls. In MCAO and Sham groups, half of the rats received a NAC stereotaxic injection whereas the other half received a cell transplantation. All the animals were sacrificed 4 weeks post-transplantation ( = 8 weeks post-MCAO). C) Representative pictures from each group of Protocol 2 are shown. (TIF) [file pone.0050444.s001.tif]

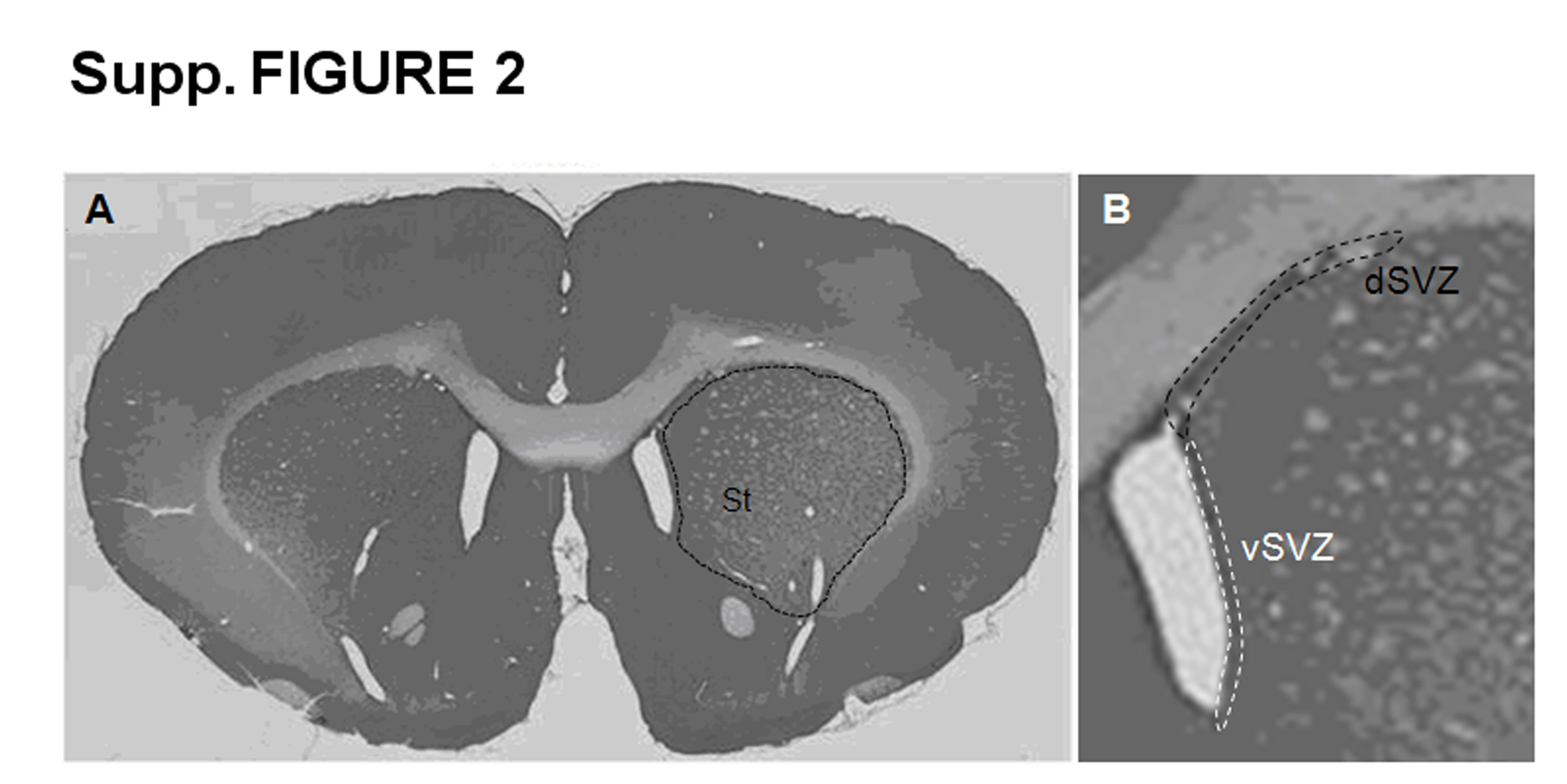

Supplement: Figure S2 — Definition of the areas of countings. A) All cells counted in the “striatal” area were contained in the dotted area. B) The dorsal SVZ (dSVZ) and ventral SVZ (vSVZ) are shown (black and white dotted lines, respectively). (TIF) [file pone.0050444.s002.tif]

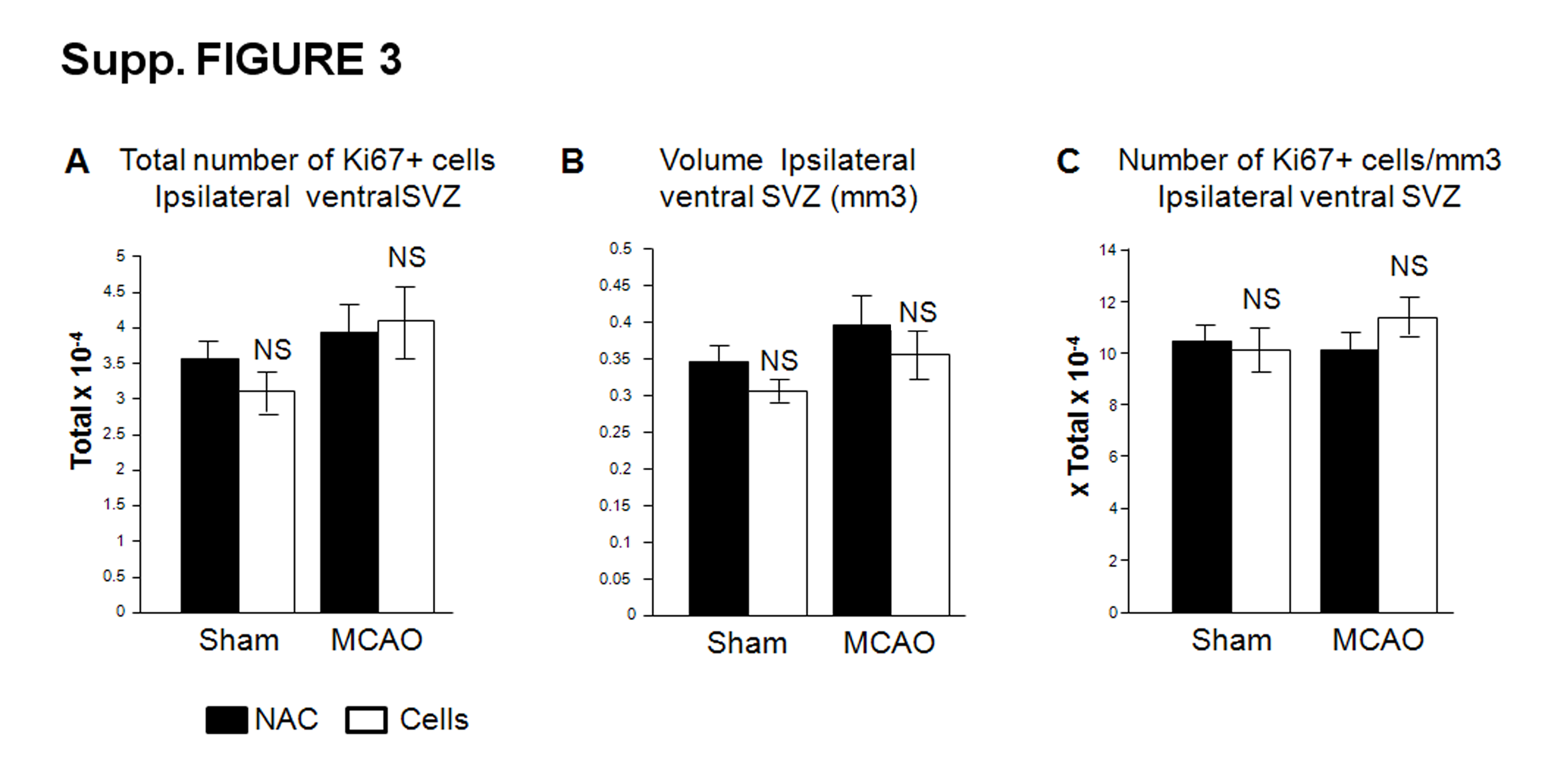

Supplement: Figure S3 — Effects of CTX0E03 on SVZ volume and proliferative activity four weeks post-transplantation. The numbers of Ki67 cells (A), the volume (B) and the density of Ki67 cells (C) in the SVZ of animals from the four groups of Protocol 2 were calculated by stereology. No difference was found in the volume (B) or proliferative activity (A and C) of the SVZ between the four groups. NS: not significant. (TIF) [file pone.0050444.s003.tif]
